# Exhaled Breath Analysis in Obstructive Sleep Apnea Syndrome: A Review of the Literature

**DOI:** 10.3390/medicina55090538

**Published:** 2019-08-27

**Authors:** Panaiotis Finamore, Simone Scarlata, Vittorio Cardaci, Raffaele Antonelli Incalzi

**Affiliations:** 1 Unit of Geriatrics, Campus Bio-Medico di Roma University, via Alvaro del Portillo 200, 00128 Rome, Italy; 2 Pulmonary Rehabilitation, IRCCS San Raffaele Pisana, 00166 Rome, Italy

**Keywords:** obstructive sleep apnea, inflammation, FeNO, exhaled breath condensate, volatile organic compounds

## Abstract

*Background and Objectives:* Obstructive sleep apnea syndrome (OSAS) represents an independent risk factor for cardiovascular, metabolic and neurological events. Polysomnography is the gold-standard for the diagnosis, however is expensive and time-consuming and not suitable for widespread use. Breath analysis is an innovative, non-invasive technique, able to provide clinically relevant information about OSAS. This systematic review was aimed to outline available evidence on the role of exhaled breath analysis in OSAS, taking into account the techniques’ level of adherence to the recently proposed technical standards. *Materials and Methods:* Articles reporting original data on exhaled breath analysis in OSAS were identified through a computerized and manual literature search and screened. Duplicate publications, case reports, case series, conference papers, expert opinions, comments, reviews and meta-analysis were excluded. *Results:* Fractional exhaled Nitric Oxide (FeNO) is higher in OSAS patients than controls, however its absolute value is within reported normal ranges. FeNO association with AHI is controversial, as well as its change after continuous positive airway pressure (C-PAP) therapy. Exhaled breath condensate (EBC) is acid in OSAS, cytokines and oxidative stress markers are elevated, they positively correlate with AHI and normalize after treatment. The analysis of volatile organic compounds (VOCs) by spectrometry or electronic nose is able to discriminate OSAS from healthy controls. The main technical issues regards the dilution of EBC and the lack of external validation in VOCs studies. *Conclusions:* Exhaled breath analysis has a promising role in the understanding of mechanisms underpinning OSAS and has demonstrated a clinical relevance in identifying individuals affected by the disease, in assessing the response to treatment and, potentially, to monitor patient’s adherence to mechanical ventilation. Albeit the majority of the technical standards proposed by the ERS committee have been followed by existing papers, further work is needed to uniform the methodology.

## 1. Introduction

Obstructive sleep apnea syndrome (OSAS) is a highly prevalent sleep breathing disorder characterized by intermittent reduction (hypopnea) and/or cessation (apnea) of airflow due to upper airways collapse and represents an independent risk factor for cardiovascular [1,2], metabolic [3], neurological diseases [4,5], and motor vehicle accidents [6]. The disease is also common in children, with a prevalence of 1–4%, and associates with behavioral and cognitive deficits [7,8]. The exact mechanism underpinning these detrimental effects is still unknown, however the pro-inflammatory state and the oxidative stress likely due to the intermittent hypoxia are deemed to play a key role [9]; indeed, the use of a continue positive airways pressure ventilation (C-PAP) has demonstrated to be effective in reducing the airways collapse, minimizing the endothelial stress and, consequently, the pro-inflammatory state [10]. Given the severity of the complications, a correct diagnosis is warranted and the gold-standard is represented by polysomnography (PSG) [11] that, however requires specialized personnel and devoted setting which limits a wide use of the tool and compels to screen the population to refer to the specialist. Questionnaires are validated screening tools, however up to 45% of patients referred with the suspicion of OSAS are not confirmed by PSG [11,12], thus new approaches in identifying patients affected by OSAS need to be identified.

Exhaled breath is abundant in volatile organic compounds (VOCs), part of which are endogenous and produced by cellular metabolism. Exhaled breath analysis, proved to detect the metabolic changes induced by OSAS, can be applied as a non-invasive tool able to shed light on the pathways modified by the disease, and also to provide a more rapid and economic instrument for diagnosis, monitoring and, eventually, characterization of the disease. Systematic reviews in this field of research are already available in literature [13,14], but, recently, several studies have been published that have enriched the available amount of evidence; furthermore, all the available reviews preceded the recently published European Respiratory Society (ERS) statement about the technical standards to follow in the exhaled breath analysis published in 2017 [15] and is therefore unclear, at the moment, to which extent the previous works adhered such methodological standards. 

The aim of this systematic review is therefore to outline the newly available evidences on the exhaled breath analysis role in OSAS, taking into account whether they conform to the proposed ERS technical standards.

## 2. Materials and Methods

**Figure 1:**
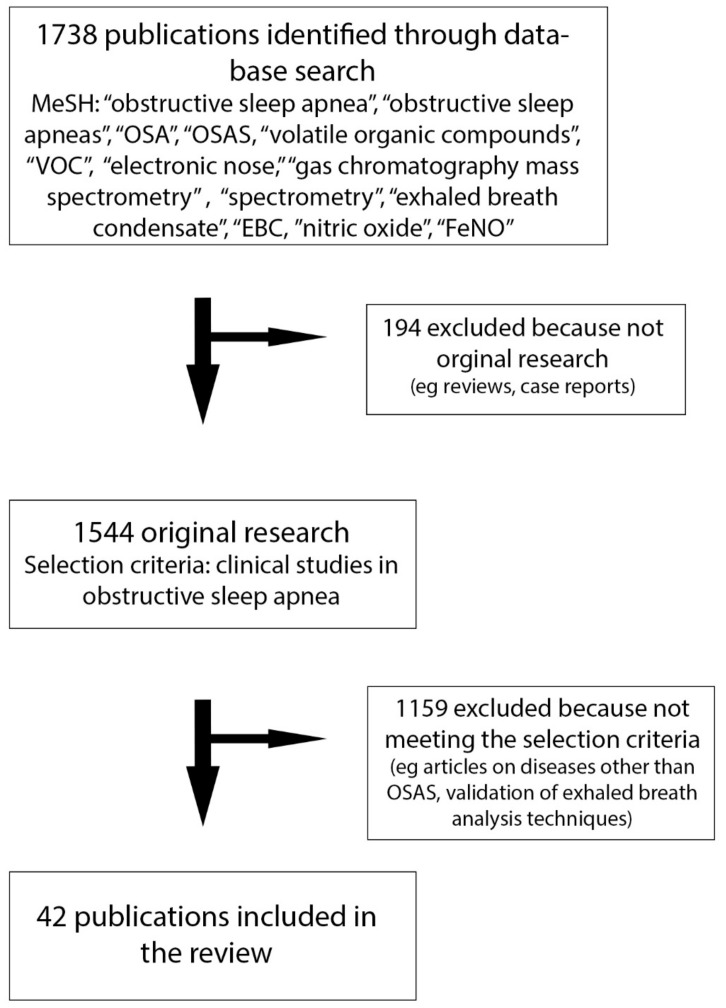
PRISMA diagram showing the flow of information through the different phases of the reviewing process.

We performed a computerized and manual literature search on PubMed, limited to English language articles published up to May 2019, to identify articles reporting original data on exhaled breath analysis in obstructive sleep apnea. We entered the following MeSH terms: Obstructive Sleep Apnea; Obstructive Sleep Apneas Syndrome; OSA; OSAS; in combination with: volatile organic compounds; VOC; electronic nose; gas chromatography mass spectrometry; spectrometry; exhaled breath condensate; EBC; nitric oxide; FeNO. Two authors (P.F. and S.S.) performed the literature search and assessed the eligibility of identified publications independently. All studies that evaluated exhaled breath analysis in OSAS were screened. Duplicate publications, case reports, case series, conference papers, expert opinions, comments, reviews and meta-analysis were excluded. The selection process is summarized in Figure 1. The literature search has been integrated with other relevant studies about methodological and clinical issues.

## 3. Results

The thirty-six studies included in the review encompass the three main domains of exhaled breath analysis: the fractional exhaled nitric oxide (FeNO), the exhaled breath condensate (EBC) and the exhaled VOCs. The characteristics of the main studies included in the review are summarized in Table 1, Table 2 and Table 3.

### 3.1. FeNO and Exhaled Carbon Monoxide (eCO)

Nitric oxide (NO) is a gaseous molecule produced by nitric oxide synthase (NOS) enzymes from L-arginine and oxygen. There are three isoforms of NOS, two are constitutively produced (endothelial NOS–eNOS– and neuronal NOS–nNOS–) and one is inducible (iNOS), increasing during inflammation [16], as that characterizing airways in asthmatic patients. Indeed, the FeNO in the gas phase emerged in the last decade of the last century as an innovative diagnostic marker of asthma [17,18]. Being non-invasive and easy to perform, FeNO raised a wide interest, allowing a deeper understanding of mechanisms underpinning its production and addressing technical issues related its measurement. Nowadays, FeNO is considered a marker of T-helper 2 cell-type inflammation, rather than a marker of asthma per se, and a marker of response to corticosteroid treatment in those patients [19].

**Figure 2:**
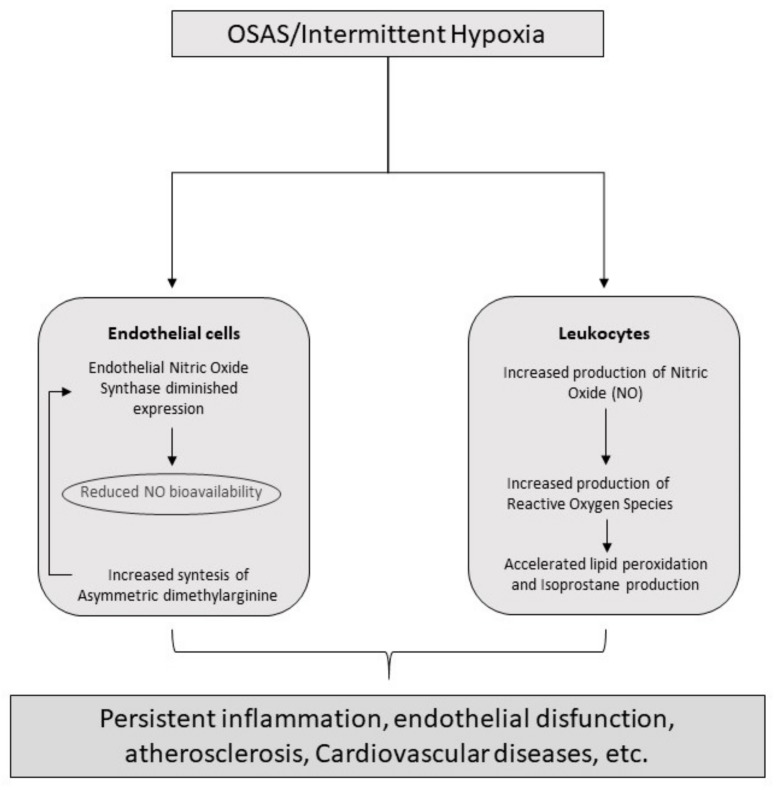
Principal inflammatory pathways induced by OSAS.

The study of FeNO in the diagnosis of OSAS has led to contradictory findings. Indeed, while some studies described a raising of FeNO level in OSAS [20,21,22,23,24,25], the majority did not confirm the finding [26,27,28,29,30] or just showed a higher concentration in OSAS patients when compared with non-obese healthy controls [31,32,33]. Besides, even considering only those studies with a positive finding, the FeNO level, albeit statistically higher than healthy controls, did not reach a clinical significance. Indeed, in all studies the mean FeNO expressed in part per billion (ppb) was below 30 ppb, which means that OSAS patients are classified in the group of individuals without airway inflammation (or without eosinophilic inflammation) or in the grey zone between 25 and 50 ppb according to the ATS guidelines [19], the same groups of healthy controls. One possible explanation of the low level of FeNO despite the inflammatory state can be the different location of the process, closer to the alveoli than the airways or in the opposite, as the result of a topical, mechanically induced inflammation at the level of the upper airway caused by snoring and apnea associated mechanical stress [34,35]. Indeed, international guidelines suggest to use a flow of 50 mL/s for the measurement of FeNO, however it is not high enough to allow the collection of the alveolar portion of NO [36]. Albeit some studies have found a statistically significant higher concentration of exhaled nitric oxide (eNO) at a flow of 250 mL/s or more in association with an elevated concentration of NO in the gas phase of Alveoli (CaNO) [22,25,37], Fortuna and colleagues reported a lower CaNO in OSAS patients than healthy controls [23] and Foresi and colleagues did not find a difference in CaNO between normotensive OSAS patients and controls [30]. The more validated hypothesis is that the increased inflammation damages the alveolar endothelium reducing the expression of the eNOS and the diffusion of NO [38]. Mechanisms of inflammation induced by OSAS are reproduced in Figure 2.

Furthermore, it is still unclear whether an overnight change in the production of eNO exists or not. While some studies reported an overnight increase in FeNO [20,24] and in the concentration of nitric oxide exhaled by the nose (nasal nitric oxide–nNO–) and by the mouth (orale nitrix oxide–oNO–) [39] in OSAS patients [20,39], other studies failed to confirm the evidence [21], or they found an overnight increase limited to subgroups of OSAS, such as obese OSAS patients [29] or children with mild OSAS but not moderate/severe [28], or healthy controls [39].

Finally, eNO has been proposed as a marker to monitor the efficacy of C-PAP therapy. Indeed, evidence in literature suggests that one-to-three month C-PAP treatment is effective in reducing FeNO [22,23,24] and increasing CaNO [23]. The effect should also be time-dependent, at least for FeNO, since a single or 2-nigth treatment with C-PAP increases CaNO [30,40] but do not reduce FeNO [30]. This suggest that C-PAP, normalizing oxygen saturation, reduces inflammation and oxidative stress, promoting alveolar endothelial function and therefore candidates CaNO as a marker of endothelial function.

Even the association of the eNO with the apnea-hypopnea index (AHI) is controversial. Indeed, while some studies found a strong and positive correlation between FeNO and AHI, with a *r* of 0.8–0.9 [23,33], or oNO and AHI (*r*: 0.46) [32] and a negative one between CaNO and AHI, with a *r* of 0.9 [23], this was not confirmed by other studies [20,21,22,27,28,39,41].

Knowledge about exhaled carbon monoxide (eCO) in OSAS is more limited than FeNO. To the extent possible, eCO has been reported higher only in severe OSAS [42], it has a weak correlation with AHI [42] and it is not normalized after one-month of C-PAP [22], probably because it needs a longer period to be normalized.

### 3.2. Exhaled Breath Condensate

**Figure 3:**
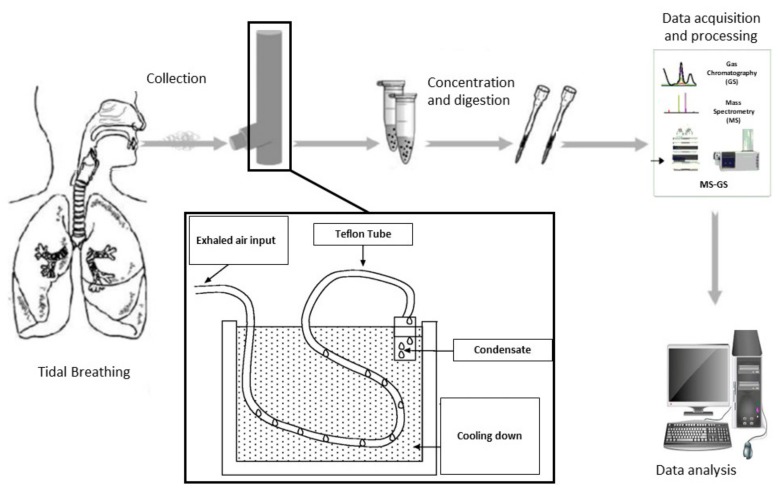
Principles of functioning of exhaled breath condensate technology.

The alveolar and airway lining fluids (ALF) contain hydrophobic and hydrophilic nonvolatile and volatile compounds which are continuously released into the environment as droplets created during breathing. In contrast to bronchoalveolar lavage, EBC is a noninvasive way to sample these compounds by directing the exhaled breath through a cooling device. The sample, mostly composed by water vapour, can be stored or immediately analyzed. Albeit noninvasive, EBC composition is highly influenced by the collection and the condenser procedure, which undermine the reliability of the achieved results. Principles of functioning of exhaled breath condensate technology is summarized in Figure 3.

#### 3.2.1. EBC pH

Given the inflammatory and pro-inflammatory state characterizing OSAS, EBC pH in OSAS was expected to be lower than healthy controls. The hypothesis has been confirmed by all the studies carried out so far, with the exception of that by Greulich and colleagues [43], with a mean absolute value of EBC pH in OSAS around 7.4, by far below the first quartile of EBC pH distribution in healthy subjects and equal to the fifth percentile [44]. pH has shown a negative correlation with AHI (*r*: −0.66), sleep time with a SaO_2_ < 90% (*r*: −0.62) and neck circumference (*r*: −0.63) [31], but also with body-mass index (BMI) (*r*: −0.54). Although Petrosyan and colleagues demonstrated that OSAS EBC pH is lower than controls, even if obese [22], the finding has not been confirmed by Carpagnano et al. [31], raising doubts about the association between EBC acidity and OSAS. Albeit it is not possible to exclude that obesity, rather than OSAS, reduces EBC pH, probably by increasing the likelihood to have gastro-esophageal reflux, it seems that EBC acidity is due to OSAS. Indeed, after the treatment with C-PAP EBC pH increases [22], becoming closer to normal reference values. A change of the EBC pH after C-PAP or surgical treatment has not been confirmed by other studies [43,45], however in both cases the EBC pH value of OSAS patients was already normal at baseline. No significant difference has been found between OSA smokers and non-smokers [46]. To conclude, all studies analyzing EBC pH performed de-aeration before the analysis, but did not performed the analysis in real time or immediately after collection without freezing or storing EBC, as suggested by international guidelines [15]. OSAS seems to increase EBC acidity, however exist a variability in the EBC pH that compels to investigate the effect of other factors.

#### 3.2.2. EBC Cytokines

EBC cytokine level has been studied in OSAS patients. As expected, all studies confirmed that the concentration of IL-6, TNF-α, IL-8 and ICAM-1 is higher than healthy controls, while IL-10 concentration, which has anti-inflammatory properties, is lower [46,47,48,49]. However, there is a wide range of cytokine concentrations among the studies: indeed, while the mean EBC IL-6 concentration was in the order of decades of pg/mL in some studies [47,48], it was below the unit in other studies [46,50], notwithstanding the concentration was expressed in the same unit of measurement. Similarly, the concentration of TNF-α in the studies of Li and colleagues [48,51] was ten times the concentration of TNF-α in the study of Antonopoulou and colleagues [46]. Hence, even pro-inflammatory cytokines seem elevated in OSAS and anti-inflammatory cytokines reduced, sampling procedure should be revised, because confounding factors, as dilution, seem to have affected the absolute value. Other confounding factors to take into account are obesity and smoking. Indeed, while some studies do not report a difference in IL-6 level between smoking and non-smoking OSAS patients [46], other studies suggest a pro-inflammatory effect of smoking [48]. Noteworthy, no doubts are on the pro-inflammatory role of obesity, with all studies confirming an elevated concentration of EBC IL-6, IL-8 and ICAM-1 in obese than normal weight individuals [47,49]. Being inflammation in OSAS closely related with intermittent hypoxia, it is not surprising that AHI was positively correlated with EBC IL-6 (*r*: 0.6−0.8) [47,48], ICAM-1 (*r*: 0.7) [49] and TNF-α (*r*: 0.85) [48] and negatively correlated with EBC IL-10 (*r*: −0.63) [51]. As expected, EBC IL-6 also positively correlated with the neck circumference (*r*: 0.5) [47]. EBC cytokines are stable over time if patients do not start a treatment [51], while effective treatment reduces their concentration. Indeed, even with different absolute values, two studies demonstrating the effectiveness of C-PAP therapy [50,51], but also the positive role of oral appliances and surgery in abating inflammation and thus EBC cytokine concentration [51].

#### 3.2.3. EBC Oxidative Stress

The EBC concentration of 8-isoprostane, a product of the lipid peroxidation of arachidonic acid and marker of oxidative stress, has been repeatedly found elevated in adult patients affected by OSAS [22,46,47,48,52,53], and in children [28]. The mean value in OSAS patients is heterogeneous, ranging from 6 to more than 30 pg/mL, and overlaps with the mean values observed in healthy controls [46,48]. Smoking seems to affect the marker concentration [48], while the role of obesity is conflicting. Indeed, while Petrosyan and colleagues found a higher level of 8-isoprostante in healthy non obese than obese individuals, both were significantly lower than OSAS patients [22], Carpagnano and colleagues observed exactly the opposite, also failing to discriminate OSAS from obese controls by 8-isoprostane concentration [47]. 8-isoprostane has shown a positive correlation with AHI, with a *r* of 0.4−0.5, [22,28,46,47,48,52,53] and neck circumference (*r*: 0.5−0.6) [47,52]. Interestingly, the concentration of 8-isoprostane is higher in the morning than in the evening in OSAS patients, with the latter similar to the concentration of healthy controls [52]. C-PAP therapy is effective in reducing the concentration of 8-isoprostane, but it is also reduced by oral appliances and surgery [50,51,52].

More limited evidence exists on the EBC concentration of hydrogen peroxide (H_2_O_2_). To the extent possible, H_2_O_2_ seems elevated in OSAS [22,54], regardless of patient’s BMI [22]. Noteworthy, obesity is associated with an increase in the H_2_O_2_ concentration in healthy controls [22]. H_2_O_2_ is also positively associated with the AHI, with the same correlation of 8-isoprostante [22], and thus with the severity of the disease, being higher in patients with moderate to severe than mild OSAS [54]. This marker is not modified by one month of C-PAP therapy [22]. While Petrosyan and colleagues clearly recommended the use of a filter on the inspiratory valve to avoid an environmental conditioning [22], it is not clear whether Malakasoti and colleagues did the same [54]. Both studies did not perform the measurement of H_2_O_2_ immediately after the collection, as suggested by the ERS guidelines [15].

#### 3.2.4. Other EBC Markers

Other markers assessed in the EBC of OSAS patients are: urates, leukotrienes and leptin. EBC concentration of acid uric, which has antioxidant capacity, has been studied in children and resulted significantly higher than healthy controls [55], probably having a role in contrasting the increased oxidative stress driven by the disease. Similarly, leukotrienes (leukotriene B4, which is also associated with the severity of the disease [22,56] and leukotriene C4/D4/E4), lipid mediators prompting inflammation, are elevated in OSAS, even though with a completely different absolute value in pg/mL among studies. Indeed, the concentration found in one study in OSAS patients completely overlaps with that found in healthy controls in another study [22,56]. Contrary to the expectations, prostaglandins (PGE2) did not show any difference between children affected by OSAS and controls [56]. Furthermore, no role seems to have leptin as an EBC biomarker of OSAS. Indeed, while obese OSAS patients have higher concentration than controls, non-obese OSAS and obese controls have the same concentration, suggesting, together with a strong and positive correlation with BMI, that obesity rather than OSAS affects the concentration of this mediator [57].

### 3.3. Volatile Organic Compounds: Spectrometry and Electronic Nose

Exhaled breath is abundant in VOCs with very low concentration, most of which are undetectable by the human nose. These molecules in part originate from the endogenous metabolism and human gut and airway microbiome [58], thus their study might provide information about any diseases threatening the internal homeostasis and thus help address their diagnosis, disease severity stratification and prognosis, as already demonstrated in other respiratory diseases [59]. To date, there exist two main approaches to the study of VOCs: the first aims to identify single biomarkers related to the disease in the mixture of molecules and it is based on the use of spectrometry, often coupled with separation techniques as gas-chromatography; the second is aimed to identify a pattern in the mixture able to discriminate, through the use of a pattern-recognition approach, the disease from other conditions and it is based on the use of electronic-noses. Both have been applied in the study of OSAS, either alone or in association. 

The use of analytical techniques have demonstrated a good accuracy in discriminating OSAS patients from healthy controls [60], even if obese [61]. However, no study has so far identified a single molecule able to discriminate OSAS from controls, thus discrimination is based on a set of VOCs. Greulich and colleagues reported in their study an increase in OSAS of 2-methylfuran, 2-(methylthio)-ethanol and hexanal and a reduction in 3-methylbutanal or 3-methylbutyraldehyde and acetone [60]. Interestingly, an increase in 2-methylfuran in serum and pharyngeal wash of those patients was also reported. However, none of the compounds described by Greulich were also identified by Dragonieri and colleagues, who reported a good discriminative capacity between OSAS and obese controls basing on the following compounds: tetrachloroethene, 2,3,5-trimethylhexane, β-pinene, 1,3,5-trimethylbenzene, 9-methylacridine, tetradecane, 6,10-dimethyl-5,9-undecadien-2-one and β-ionone [61]. Besides, Aoki and colleagues found that although almost all the aromatic and satured hydrocarbons are more expressed in the exhaled breath of severe OSAS patients, only isoprene is always elevated in OSAS, regardless the severity of the disease [62].

**Figure 4:**
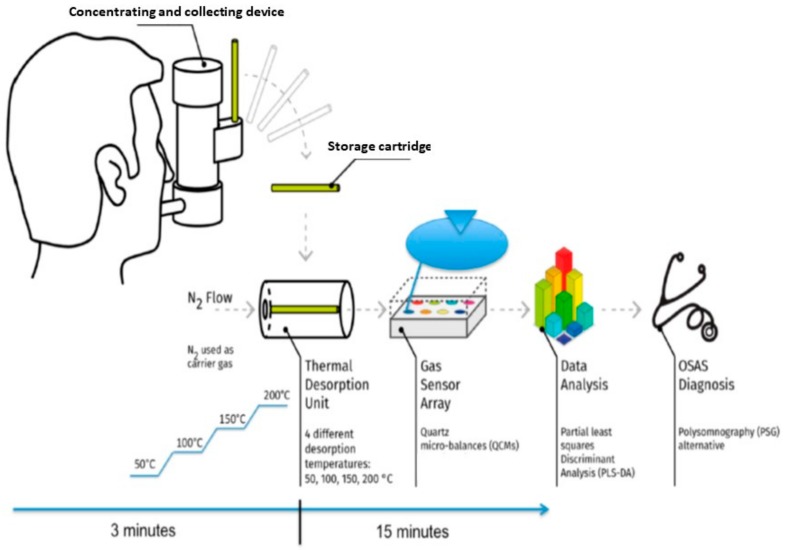
Measure chain of an e-nose based sensor system.

A good discriminative accuracy in discriminating OSAS from normal weight controls and chronic obstructive pulmonary disease (COPD) patients has also been demonstrated by the use of electronic noses, which showed a lower accuracy in discriminating people affected by the disease from healthy obese controls [43,63,64,65]. As already observed for other exhaled breath markers (e.g., 8-isoprostane), the breath pattern changed overnight in OSAS patients but not in controls, likely due to the inflammation and oxidative stress promoted by the intermittent hypoxia; indeed there was a difference in breath pattern between OSAS and controls only in the morning. Noteworthy, the difference is still present after the exclusion of patients suffering from gastro-esophageal reflux and COPD [66]. The finding is in line with that of Olopade and colleagues who reported a higher concentration of oral pentane in the morning than in the evening [39]. While some studies found a positive correlation between the breath pattern and the AHI [43], other studies failed to confirm the finding [66]. Albeit apparently contradictory, it is possible that the association between AHI and breath pattern is mediated by patients’ comorbidities, as suggested by Incalzi and colleagues [67]. Breath-pattern is sensitive to the effects of the C-PAP therapy, indeed concentrations of isoprene and acetone decrease [62] and it is possible to discriminate treated and untreated patients with good accuracy [68]; even a single night treatment is associated with a change in the breath pattern. Interestingly, the breath pattern change does not have the same characteristics in all OSAS patients, with two different types of response being distinguished depending on the comorbidities of those individuals [67]. Noteworthily, almost all the studies did not perform an external validation of the discriminative model, hence it is not possible to exclude an overfitting of the models, even though minimized by the use of internal cross-validation. Technical and operative descriptions of these approaches have been summarized in Figure 4 and discussed in detail elsewhere [69,70].

## 4. Discussion

This updated systematic review confirms the promising role of exhaled breath analysis in the understanding of the mechanisms underpinning disease and its clinical relevance in identifying individuals affected by OSAS. Besides, in addition to previous reviews of the field, it shows that, although the majority of the technical standards proposed by the ERS committee have been followed, more research is needed to stadardize the methodology and hence reduce the variability in the results.

OSAS is characterized by an endothelial dysfunction, arterial stiffening and elevated levels of inflammatory markers as an effect of the intermittent hypoxia caused by the upper airways collapse [71] which increase the risk to develop cardiovascular, metabolic or neurological events. Indeed, hypoxia increases the production of reactive oxygen species (ROS) and thus the oxidative stress, which impairs the phosphorylation of NOS [72,73], reduces the release of nitric oxide and promotes the endothelial dysfunction. Results of studies on FeNO are in line with this notion. Indeed, overall the concentration of FeNO measured at a flow of 50 mL/s is below the 50 ppb, identified by the American Thoracic Society (ATS) as a threshold of the presence of eosinophil airway inflammation. Moreover, the reduced CaNO in the studies of Fortuna and Foresi and its elevation after effective treatment support the existence of an alveolar damage in the disease [23,30]. Furthermore, intermittent hypoxia fosters the development of a chronic inflammation, and this is confirmed by the studies carried out on the EBC. Indeed, pro-inflammatory cytokines increase while anti-inflammatory cytokines decrease in the breath of those patients, and the markers of oxidative stress are elevated in the morning [39,52], as demonstrated also by the studies on the breath pattern [66]. Moreover, inflammatory cells were increased in the muscular layer of patients with OSAS, with CD4+ and activated CD25+ T cells (both increased approximately threefold) predominating. Inflammation was also present in upper airway (UA) mucosa, but with a different pattern consisting of CD8+ (2.8-fold increase) and activated CD25+ (3.2-fold increase) T cell predominance, suggesting that inflammatory cell infiltration affects not only the mucosa, but also the UA muscle of patients with OSAS, this potentially leading to a systemic pro-inflammatory spillover of cytokines and mediators that could promote and amplify chronic inflammatory response [35]. Indeed, these proposed mechanisms are still far from being confirmed and further research is needed to confirm this pathophysiologic mechanism.

Although all the techniques studying volatile and non-volatile compounds are able to discriminate OSAS patients from controls, EBC and the study of volatile organic compounds seem more promising than FeNO for a clinical use. However, efforts are needed to address some the technical and non-technical issues that are hindering the applicability of breath analysis in clinical practice. The role of smoking in increasing inflammation, as well as that of obesity, should be deeper investigated in the studies about OSAS. Besides, issues as the dilution of the EBC [74] or the lack of external validity in most of the studies about volatile organic compounds need to be addressed to increase the reliability of the techniques.

Breathprint analysis of VOCs might have practical applications and could act as a valuable instrument in OSAS management in the next future: considering the high prevalence of OSAS in the general population and its dramatic impact on health status, any effort should be made in order to detect and treat it as soon as possible. Breathprint analysis might complement, or even replace questionnaires in the screening process and, consequently, improve the cost/effectiveness ratio of polysomnography. Furthermore, VOCs analysis could be used to monitor the response to, and the adherence with C-PAP ventilation [57]. Finally, the breath print analysis could help better understanding of the heterogeneity of OSAS phenotypes [69] and define their prognosis, as in other respiratory diseases [75].

## 5. Conclusions

To conclude, in the era of precision medicine breath analysis, being non-invasiveness, rapid and economic, might play a key role in the understanding of the pathways underpinning OSAS and in the clinical management of the patients affected by the disease. 

## Figures and Tables

**Table 1:** Exhaled nitric oxide for the diagnosis of OSAS patients.

First Author (Year) [Reference]	OSAS	AHI	NO	Device	FeNO ppb	HC	NO ppb	*p*-Value
Zhang (2018) [20]	75	28.1 e/h	FeNO (1)	NIOX MINO^®^	(1) 21.08 (8.79)	30	(1) 16.9 (6.86)	0.02
			nNO (2)	50 mL/s	(2) 487 (115.8)		(2) 413 (73.1)	
Przybylowski (2006) [21]	66	40.3 e/h	FeNO	CA 45−55	23.1 (14.8)	53	16.8 (9.8)	<0.05
Petrosyan (2008) [22]	26	63.7 e/h	FeNO (1)	LR2000 CA 250 mL/s	(1) 7.1 (4.6)	9 O * 10NO †	(1) 5 (1.1) * (1) 4.2 (1.9) (2) 366 (169) * (2) 539 (264) † (3) 4.8 (1) * (3) 4.7 (1.2) †	<0.05 <0.05 <0.01 NS <0.05 <0.05
			nNO (2)		(2) 610 (222)			
			eCO (3)		(3) 6.4 (2.9)			
Olopade (1997) [39]	16	47.7 e/h	FeNO (1)	CA NA	(1) 6.6 (0.8)	8	(1) 6.8 (1.3)	NA
			nNO					
JalilMirmohammadi (2014) [29]	31 O *	39.5 e/h	FeNO	NObreath^®^	14.1, 3–31 *	7	22.1, 5–58	NS
	16 NO †	40.1 e/h		50 mL/s	15.8, 2–31 †			
Gut (2016) [41]	28	6.6 e/h	nNO	Eco Medics AG	867 (371)	23	644 (166)	0.047
Fortuna (2011) [23]	30	NA	FeNO (1)	NIOX	(1) 27.2 (18)	30	(1) 16.7 (8)	0.0006
		>15 e/h	CaNO (2)	50 mL/s				
Foresi (2007) [30]	34	31.3 e/h	FeNO	NOA 280 50,120,190, 250 e 300 mL/s	21.8 (1.9)	9	15.4 (1.7)	NS
Duarte (2019) [26]	199	30.1 e/h	FeNO	NIOX MINO^®^ 50 mL/s	20.2 (14.5)	30	16.9 (10.6)	0.221
Depalo (2008) [33]	18 O	59.1 e/h	FeNO (1)	CA	(1) 23.1 (2.1)	15 O *	(1) 17.9 (2.1) *	NS
			iNOS (2)	45 mL/s		10NO †	(1) 7.2 (0.6) †	<0.001
Culla (2010) [32]	39	NA	FeNO (1)	CA	(1) 23.1, 19−28	26 AS * 15 CR † 24 ‡	(1) 40, 32−50 * (1) 22, 16−32 † (1) 11, 8−14 ‡ (2) 71, 56−91 * (2) 54, 40−73 † (2) 63, 59−73 ‡	NS NS <0.001 0.015 0.009 <0.001
		>10 e/h	oNO (2)	50 mL/s	(2) 104, 80−135			
Carpagnano (2008) [31]	30 O	59.1 e/h	FeNO	CA	31.6 (1.6)	20 O *	27.1 (1.8) *	NS
				45 mL/s		10 NO †	4.8 (0.7) †	<0.001
Duong-Quoy (2015) [25]	52	25.6 e/h	FeNO (1)	FeNO+ 50,100,150,350 mL/s	(1) 16.7 (11.4) (2) 4 (1.7)	30	(1) 9.4 (6.6)	0.003
			CaNO (2)				(2) 2.2 (0.7)	0.001
Barreto (2018) [28]	17 CH mild * 17 CH mod/sev †	2.3 e/h	FeNO	HyPair FENO	11, 7.9−14.8 *	20	13.5, 8.7−19.9	NS
		8.6 e/h		50 mL/s	10, 6.5−16 †			
Agustì (1999) [27]	24	55 e/h	FeNO	CA	22.2 (3)	7	19.7 (3.2)	NS
				NA				
Chua (2013) [24]	75	40 e/h	FeNO	NIOX MINO^®^	13.4 (6.5)	29	6.5 (3.5)	<0.001
				50 mL/s				

For those studies analyzing the change of FeNO during the night, the mean (SD) is that before the night. Legend: OSAS: obstructive sleep apnea syndrome; AHI: apnea-hypopnea index (e/h: events per hour); HC: healthy controls; FeNO: fractional exhaled nitric oxide; nNO: nasal nitric oxide; oNO: oral nitric oxide; O: obese; NO: not obese; CA: chemiluminenscence analyser; AS: asthma; CR: chronic rhinitis/rhinosinusitis. Symbols (*,†,‡) are used to link the value with the subgroup.

**Table 2:** Exhaled breath condensate for the diagnosis of OSAS.

First Author (Year) [Reference]	OSAS	AHI	Molecule	Standards	Value	HC	Value	*p*-Value
Carpagnano (2008) [31]	30 OS	59.1 e/h	pH	Volume collection **X** Tidal breathing ✔ Nose clip ✔ Storage ✔ Deaeration ✔	7.48 (0.07)	20 ON * 10 NO †	7.68 (0.08) * 7.99 (0.03) †	NS <0.01
Carpagnano (2003) [52]	18	59.2 e/h	8-Isoprost.	Volume collection **X** Tidal breathing ✔ Nose clip ✔ Storage ✔	9.5 (1.9) pg/mL	12	6.7 (0.2) pg/mL	<0.001
Petrosyan (2008) [22]	26	63.7 e/h	pH (1)	Volume collection ✔ Tidal breathing ✔ Nose clip ✔ Deaeration ✔ Storage ✔	(1) 7.2 (0.69)	9 O * 10NO †	(1) 7.79 (0.09) * (1) 7.77 (0.05) † (2) 4 (0.2) pg/mL * (2) 5 (1.9) pg/mL † (3) NA * (3) NA † (4) 1.2 (0.9) uM * (4) 0.3 (0.4) uM †	<0.01 <0.01 <0.001 <0.001 <0.001 <0.001 <0.05 <0.01
			8-Isoprost.(2)		(2) 12 (6) pg/mL			
			Leuk.B4 (3)		(3) 8 (6) pg/mL			
			H2O2 (4)		(4) 5.8 (8.9) uM			
Vlasic (2011) ▲ [55]	17	3.54 e/h	Urates	Volume collection ✔ Tidal breathing ✔ Nose clip ✔ Storage **X**	86, 28−113 µmol/L	12	31, 23−42 µmol/L	0.046
Malakasioti (2012) ▲ [54]	12 Mo-S (1) 22 Mild (2)	13.6 e/h 2.8 e/h	log(H2O2)	Volume collection ✔ Tidal breathing ✔ Nose clip ✔ Storage ✔	0.4 (1.1) −0.9 (1.3)	16	−1.2 (1.2)	(1vs3) 0.003 (1vs2) 0.015
Li (2009) [48]	22 Mild * 22 Mo † 24 S ‡	14.1 e/h 29.7 e/h 70.1 e/h	8-Isoprost.(1) IL−6 (2) TNF−α (3) IL−10 (4)	Volume collection **X** Tidal breathing ✔ Nose clip ✔ Storage ✔	(1) 15.5 (2) pg/mL * (1) 18.8 (2) pg/mL † (1) 21.8 (2) pg/mL ‡ (2) 8.4 (1) pg/mL * (2) 13.9 (2) pg/mL † (2) 15.5 (2) pg/mL ‡ (3) 96.1 (8) pg/mL * (3) 116 (11) pg/mL † (3) 128.2 (8) pg/mL ‡ (4) 48.2 (6) pg/mL * (4) 31.2 (5) pg/mL † (4) 24 (4) pg/mL ‡	22 HNS ҂ 10 HS ‖	(1) 12.6 (2) pg/mL ҂ (1) 16.8 (2) pg/mL ‖ (2) 6.8 (1) pg/mL ҂ (2) 10.9 (2) pg/mL ‖ (3) 83.7 (4) pg/mL ҂ (3) 97 (6) pg/mL ‖ (4) 56.8 (7) pg/mL ҂ (4) 38.6 (7) pg/mL ‖	(1) <0.001 (2) <0.001 (3) <0.001 (4) <0.001
Carpagnano (2002) [47]	18	59.2 e/h	8-Isoprost.(1) IL-6 (2)	Volume collection **X** Tidal breathing ✔ Nose clip **X** Storage ✔	(1) 7.4 (0.7) pg/mL (2) 8.7 (0.3) pg/mL	10 ON * 15 NO †	(1) 5 (0.3) pg/mL * (1) 4.5 (1) pg/mL † (2) 2.1(0.2) pg/m *l (2) 1.6(0.1) pg/mL †	0.4 <0.005 <0.05 <0.001
Goldbart (2006) ▲ [56]	29 Mild * 21 Mo-S †	< 5 e/h > 5 e/h	Leuk.B4 (1) LeukTC4/D4/E4 (2) PGE2 (3)	Volume collection **X** Tidal breathing ✔ Nose clip **X** Storage ✔	(1) 66.4 (4) pg/mL * (1) 97.6 (6) pg/mL † (2) 27.6 (8) pg/mL * (2) 45.1 (11) pg/mL † (3) ≈ 29 pg/mL * (3) ≈ 35 pg/mL †	NA	(1) 27.8 (4) pg/mL (2) 15.7 (8) pg/mL (3) ≈ 19 pg/mL	<0.001 <0.001 NS
Carpagnano (S 2010) [57]	36 OS * 28 NOS †	57.6 e/h 40.8 e/h	Leptin	Volume collection **X** Tidal breathing ✔ Nose clip ✔ Storage ✔	5.12, 3.8−6.6 ng/mL * 4.1, 3.9−5.2 ng/mL †	24 ON ‡ 20 NO ҂	4.2, 3.6−5 ng/mL ‡ 3.2, 2.4−4 ng/mL ҂	<0.05
Barreto (2018) ▲ [28]	17 CH mild * 17 CH Mo-S †	2.3 e/h 8.6 e/h	8-Isoprost.	Volume collection **X** Tidal breathing ✔ Nose clip ✔ Storage ✔	45, 30−88 pg/mL * 52, 39−130 pg/mL †	20	19.2, 12−32 pg/mL	<0.01 <0.01
Antonopoulou (2008) [46]	45	39 e/h	pH (1) 8-Isoprost.(2) IL-6 (3) TNF-α (4)	Volume collection **X** Tidal breathing ✔ Nose clip? Storage ✔ Deaeration ?	(1) 7.44 (0.2) (2) 30.5 (19) pg/mL (3) 0.53 (0.3) pg/mL (4) 1.4 (0.9) pg/mL	25	(1) 7.46 (0.1) (2) 12 (3) pg/mL (3) 0.21 (0) pg/mL (4) 0.6 (0.3)pg/mL	0.0009 <0.0001 0.03 0.0002
Carpagnano (J 2010) [49]	12 OS * 10 NO †	48.8 e/h	IL-8 (1) ICAM-1 (2)	Volume collection **X** Tidal breathing ✔ Nose clip ✔ Storage ✔	(1) 17.5 (2) pg/mL * (1) 14.8 (1.9) pg/mL † (2) 100 (3.6) pg/mL * (2) 88.6 (3.9) pg/mL †	10 ON 8 NO	(1) 17 (0.7) pg/mL * (1) 7 (0.5) pg/mL † (2) 93 (2.6) pg/mL * (2) 51 (1.2) pg/mL †	NS <0.001 NS <0.001
Karamanli (2014) [50]	35 C-PAP	3.8 vs. 45.6	8−Isoprost. (1) IL-6 (2) TNF-α (3) Peroxynitr.(4)	Volume collection **X** Tidal breathing ✔ Nose clip ✔ Storage ✔	(1) 3 vs. 5.7 pg/mL (2) 0.3 vs. 1.1 pg/mL (3) 26.8 vs. 29 pg/mL (4) 4.6 vs. 17.3 pg/mL	-	-	0.027 <0.001 <0.001 0.037
Li (2008) [51]	33 C-PAP† 28 UNT ‡ 2 OrAp ⁕ 5 SURG ҂ 22 HC	24.7 vs. 45.7 32.5 vs. 31.4 12.9 vs. 38.6 28.8 vs. 32.7	8−Isoprost. (1) IL−6 (2) TNF−α (3) IL-10 (4)	Volume collection **X** Tidal breathing ✔ Nose clip ✔ Storage ✔	(1) 15 vs. 20 pg/mL † (1) 17 vs. 17 pg/mL ‡(1) 12 vs. 18 pg/mL * (1) 13 vs. 20 pg/mL ҂ (2) 10 vs. 14 pg/mL † (2) 11 vs. 11 pg/mL ‡ (2) 8 vs. 11 pg/mL * (2) 9 vs. 13 pg/mL ҂	(3) 97 vs. 118 pg/mL † (3) 108 vs. 108 pg/mL ‡ (3) 105 vs. 119 pg/mL * (3) 88 vs. 117 pg/mL ҂ (4) 42 vs. 21 pg/mL † (4) 38 vs. 38 pg/mL ‡ (4) 37 vs. 35 pg/mL * (4) 50 vs. 31 pg/mL ҂		Unknown

▲ Study carried out in children. Legend: OSAS: obstructive sleep apnea syndrome; AHI: apnea-hypopnea index (e/h: events per hour); HC: healthy controls; Mild: Mo-S: moderate-severe; OS: obese OSAS; NOS: non-obese OSAS; ON: obese healthy controls; NO: non-obese healthy controls; C-PAP: continuous positive airway pressure; UNT: untreated; OrAp: oral appliances; SURG: surgery. ✔: technical standard satisfied; **X:** technical standard not satisfied. Symbols (*,†,‡,҂) are used to link the value with the subgroup.

**Table 3:** Volatile organic compounds analysis for the diagnosis of OSAS patients.

First Author (Year) [Reference]	OSAS	AHI	Device	Standards	Controls	Discriminative capacity	*p*-Value
Greulich (2013) [43]	40	33.6 e/h	E-nose (Cyranose320) *Disposable bags*	Internal cross-validation ✔ External validation set ✔	20	AUROC 0.85 (95%CI 0.74−0.96)	-
Dragonieri (2016) [65]	13 (6 validation set)	44.8 e/h	E-nose (Cyranose320) *Disposable bags*	Internal cross-validation ✔ External validation set ✔	15 COPD (6 validation set) 13 OVS. (6 validation set)	AUROC OSAS vs. OVS.: 1 AUROC OSAS vs. COPD: 0.83	<0.001 <0.01
Kunos (2015) [66]	17 OSAS 9 habitual snorers	29.8 e/h	E-nose *Mylar bags*	Internal cross-validation ✔ External validation set **X**	10	Accuracy OSAS vs. HC (morning): 77%	<0.001
Antonelli Incalzi (2015) [67]	50 C-PAP	41.8 e/h	E-nose (BIONOTE) *Pneumopipe + TenaxGR*	Internal cross-validation ✔ External validation set **X**		29 consonant change 21 discordant change	
Dragonieri (2015) [61]	19 OS	27.8 e/h	GC-MS (1) E-nose (2) (Cyranose320) *Tedlar bags* *Carboxen and Carbopack cartridges*	Internal cross-validation ✔ External validation set **X**	14 ON 20 NO	(1) Accuracy OS vs. ON: 91% (2) AUC OS vs. NO: 1 (2) AUC OS vs. ON:0.7	
Scarlata (2017) [63]	20 hypo 20 non-hypo	13.6 e/h 2.8 e/h	E-nose (BIONOTE) *Pneumopipe + TenaxGR*	Internal cross-validation ✔ External validation set **X**	56 NO 20 non-hypo COPD 20 ON	Accuracy OSA vs. HC: 0.99 Accuracy OSAS vs. COPD: 0.75	
Benedek (2013) [64]	18	2 e/h	E-nose (Cyranose320) *Mylar bags*	Internal cross-validation **X** External validation set **X**	10 habitual snoring	AUROC: 0.84	<0.003
Greulich (2018) [60]	15	26 e/h	Ion mobility mass spectrometry (1) E-nose (Cyranose320) (2)	Internal cross-validation ✔ External validation set **X**	15	(1) AUROC 0.79 (2) AUROC 0.9	0.004 <0.001

Legend: OSAS: obstructive sleep apnea syndrome; AHI: apnea-hypopnea index (e/h: events per hour); AUROC: Area under receiver operating curve; COPD: chronic obstructive pulmonary disease; OVS.: overlap syndrome; C-PAP: continuous positive airway pressure; OS: obese OSAS; ON: obese healthy controls; NO: non-obese healthy controls; hypo: hypoxemic; non-hypo: non hypoxemic; ✔: technical standard satisfied; **X:** technical standard not satisfied.
